# A pragmatic clinical effectiveness trial of a novel alternative to punishment for school-based substance use infractions: study protocol for the iDECIDE curriculum

**DOI:** 10.3389/fpubh.2023.1203558

**Published:** 2023-08-21

**Authors:** Caroline A. Gray, Vanessa Iroegbulem, Brooklyn Deming, Rebecca Butler, Dan Howell, Michael P. Pascale, Alec Bodolay, Kevin Potter, Amy Turncliff, Stacey Lynch, Jennie Whittaker, Julia Ward, Devin Maximus, Gladys N. Pachas, Randi M. Schuster

**Affiliations:** ^1^Department of Psychiatry, Center for Addiction Medicine, Massachusetts General Hospital, Boston, MA, United States; ^2^MassHealth Office of Behavioral Health, Boston, MA, United States; ^3^Massachusetts Department of Public Health, Office of Youth and Young Adult Services, Boston, MA, United States; ^4^Rockfern Scientific, Ashland, MA, United States; ^5^Institute for Health and Recovery, Watertown, MA, United States; ^6^Harvard Medical School, Boston, MA, United States

**Keywords:** school, substance use, diversion programs, alternatives to punishment, prevention, equity

## Abstract

**Background:**

Adolescents who use alcohol and other drugs on school campuses are at heightened risk for adverse consequences to their health and wellbeing. Schools have historically turned to punitive approaches as a first-line response to substance use. However, punishment is an ineffective deterrent for substance use and may cause harm and increase inequities. iDECIDE (Drug Education Curriculum: Intervention, Diversion, and Empowerment) was developed as a scalable and youth-centered drug education and diversion program that can be used as a skills-based alternative to punishment. We aim to evaluate the effectiveness of the iDECIDE curriculum as an alternative to punishment (ATP) for school-based substance use infractions in the context of a large pragmatic clinical effectiveness study.

**Methods:**

We will conduct a Type 1, hybrid effectiveness-implementation trial. Using a stepped wedge design with approximately 90 middle and high schools in Massachusetts, we will randomly allocate the timing of implementation of the iDECIDE curriculum compared to standard disciplinary response over approximately 36 months. We will test the overarching hypothesis that student-level outcomes (knowledge of drug effects and attitudes about substance use; frequency of substance use; school connectedness) improve over time as schools transition from a standard disciplinary response to having access to iDECIDE. The secondary aims of this trial are to (1) explore whether change in student-level outcomes vary according to baseline substance use, number of peers who use alcohol or other drugs, age, gender, and school urbanicity, and (2) determine the acceptability and feasibility of the iDECIDE curriculum through qualitative stakeholder interviews.

**Discussion:**

Substance use continues to be a major and rapidly evolving problem in schools. The importance of moving away from punishment to more restorative approaches is widely accepted; however, scalable alternatives have not yet been identified. This will be the first study to our knowledge to systematically evaluate an ATP for students who violate the school substance use policy and is well poised to have important implications for policy making.

## 1. Introduction

In 2022 ~7%, 13%, and 22% of 8th, 10th, and 12th-grade students, respectively, in the U.S. reported using any illicit substance in the past 30 days (1). Middle and high school campuses have become a common point of access for alcohol and other drugs. According to the Massachusetts Department of Elementary and Secondary Education (DESE), rates of chemical health violations increased 27% in the 2 years leading up to COVID-19 quarantine (2). This rise in at-school substance use is in part due to the advent of electronic cigarettes and other vaping devices that have become increasingly popular, inconspicuous, and concealable from peers and school personnel (3). Compared with out-of-school use, in-school substance use is associated with increased odds of intoxicated driving, fighting, weapon-carrying at school, risky sexual behavior, sexual assault, intimate partner violence, depression, suicidal ideation, and attempted suicide (4). It is thus a priority for schools to define effective responses to this emerging problem to mitigate risk for adverse consequences to health and wellbeing.

Schools have historically relied on exclusionary, punitive responses (e.g., detention, suspension, expulsion) to address violations to school substance use policy. However, punitive approaches are not only ineffective deterrents for substance use (5–7), but may also increase risk for substance use escalation (8, 9), academic difficulties (9–11), disengagement from school (10), and delinquency (12, 13). Moreover, being suspended only once in 9th grade increases risk of drop-out by three-fold (14–16). There are several pathways by which punishment can catalyze negative outcomes, including increased unsupervised time, alienation from peers, reactivity, and disconnection from school supports, resources, and services (17, 18). Punitive responses may also stigmatize and label students, with possible effects including deteriation of trust and breakdown of student-school relationships (19). Supportive, alternative to punishment (ATP) responses to youth substance use infractions provide greater opportunities for students to build positive relationships with a trusting, caring adult at school, improving school connectedness and possibly reducing future infraction rates (20). Finally, punitive responses are a missed opportunity for early intervention as they fail to address the multitude of factors that may lead an adolescent to initiate or escalate substance use (e.g., attempt to fit in with peers, manage internalizing symptoms, avoid environmental stressors) (3). Supportive ATP, like diversion programs, can uncover underlying motivations for substance use and align goals with core values, increasing the likelihood of lasting behavior change.

To determine the need for ATPs in middle and high schools across Massachusetts, this study team conducted a statewide survey in May-June 2020 of school stakeholders, including district administrators, principals and vice principals, school resource officers, guidance counselors, and nurses (21). The survey asked about beliefs, attitudes, and actions that schools take regarding school-based substance use infractions as well as perceived barriers to implementing diversion programs. Most stakeholders reported that while the most common response to substance use in their school/district included some sort of punishment (85.3%), ATPs including diversion programs were perceived to be more effective than punishment. Multiple barriers to implementation of ATPs were identified including lack of availability of curriculum that address substances beyond nicotine. These preliminary data highlight widespread interest in but limited access to evidence-based ATPs.

Availability of scalable, sustainable, and evidence-based ATPs may also be an important step in bridging racial disparities, as Black, Indigenous, and other Youth of Color (BIYOC) and those in resource-limited settings are disproportionately impacted by punitive approaches for school-based substance use infractions (11, 13). According to the 2017–2018 data from the U.S. Department of Education's Office of Civil Rights, Black students were suspended almost three times as often as white students and were referred to law enforcement more than two times as often for infractions that took place on school campuses (22). Meaningful steps are urgently needed to limit the inequitable use of punishment for substance use at school to improve individual- and community-level outcomes, interrupt the school-to-prison pipeline, and begin to dismantle sources of structural racism.

The primary aim of this study is to evaluate the effectiveness of iDECIDE (Drug Education Curriculum: Intervention, Diversion, and Empowerment), a novel, free, state-funded (Massachusetts Department of Public Health, MA DPH) substance use diversion program in the context of a large pragmatic clinical effectiveness study. We will test the overarching hypothesis that student-level outcomes (i.e., knowledge of drug effects, attitudes about substance use, frequency of substance use, and school connectedness) improve over time as schools transition from standard disciplinary responses to having access to iDECIDE, an educational and therapeutic ATP. Secondary aims of this trial are to (1) explore whether change in student-level outcomes varies according to baseline substance use, number of peers who use alcohol or other drugs, age, sex, gender, and school urbanicity, and (2) determine the acceptability and feasibility of the iDECIDE curriculum as an ATP for school-based substance use infractions.

## 2. Materials and methods

### 2.1. Design

**Figure 1:**
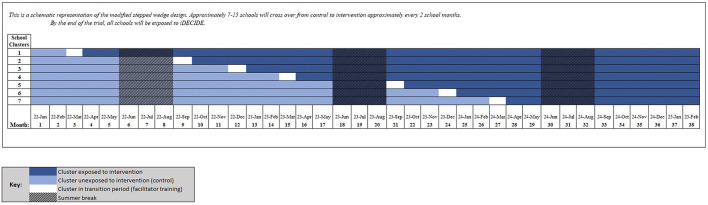
Stepped wedge schema.

We will conduct a Type 1, hybrid effectiveness-implementation trial (23) guided by Proctor's implementation model (24). Using a stepped wedge design with approximately 90 schools, we will randomly allocate the timing of implementation of the iDECIDE curriculum compared to standard disciplinary response, over approximately 36 months. Timing of curriculum implementation will be staggered over seven clusters, with each cluster composed of ~7–15 schools (size of clusters accounting for districts randomized together). One cluster of schools will cross from control (unexposed phase) to intervention (exposed phase) approximately every two to three school months until all schools are exposed to iDECIDE. This design will allow transition periods for training of school-based facilitators, during which clusters will not be considered as either in the control (unexposed) or intervention (exposed) phase of the study. See Figure 1 for the approximate stepped wedge schema.

Data collection will occur during both unexposed and exposed study phases. School staff will refer students with school-based substance use infractions to the study team as close to the time of infraction as possible through an online, secure referral system. Referrals will be submitted while schools are in both unexposed and exposed study phases. Once referred to the study team, students will be given the opportunity to enroll in a three visit study. Baseline visits will occur as close as possible to the receipt of school referrals to establish information most representative of thoughts, feelings, and behaviors at the time of the initial infraction. Follow-up visits will occur ~45 and 90 days after the baseline visit to ascertain thoughts, feelings, and behaviors occurring after the time of the school response to the substance use infraction, which may or may not involve iDECIDE depending on whether the school is in the unexposed or exposed phase of the study. Study procedures will take place remotely, at the student's school, in a private space at a local public library, or at the Massachusetts General Hospital (MGH) lab based on the preferences of the school, student, and/or parent/guardian.

This trial has been approved by the Mass General Brigham (MGB) Institutional Review Board (IRB). The trial will be conducted in accordance with the Consolidated Standards for Reporting Trials (CONSORT) statement (25–28) and will be reported here in accordance with the Standard Protocol Items: Recommendations for Intervention Trials (SPIRIT) statement (29, 30).

### 2.2. Partnering schools

Middle and high schools across all 351 municipalities in Massachusetts were given the opportunity to participate in this trial. Proactive school recruitment occurred across a period of approximately 12 months. Schools had the opportunity to attend an informational session about iDECIDE and/or meet one-on-one with members of the evaluation team to discuss study involvement. Recruitment for schools occurred through state listservs, word of mouth, and facilitated introductions through DPH, local community coalitions, and other community agencies. Inclusion criteria for schools include (1) middle or high school, serving grades 6-12, (2) agreement to refer students with a school-based substance use infraction to the MGH evaluation team (unless opted out by the student's parent/legal guardian; see section on Consent or Assent below), and (3) a signed letter of commitment from the appropriate school/district representative.

The first school cohort was enrolled in January 2022 (Cohort 1; *n*_schools_ = 69 schools). Due to substantial unsolicited interest in the curriculum from schools following the return to in-person learning after the COVID-19 pandemic, one additional wave of schools was onboarded and separately randomized in September 2022 (Cohort 2; *n*_schools_ = 33 schools).

### 2.3. Participants and recruitment

Participants will be recruited by direct school referral only. Parents/guardians will have the opportunity to opt their students out of the referral process at the beginning of each school year. Upon receipt of a school referral, the MGH evaluation team will invite the student to participate in the trial. Students will be eligible to participate if they were not opted out by the parent/guardian, recently referred for a substance use-related infraction at school or at a school-sanctioned event, are able to read and write comfortably in English, Spanish, or Portuguese, and are able to safely participate in the protocol in the opinion of the investigator. Participants will be reimbursed $50 per visit plus bonuses for urine samples for toxicology testing ($10/per sample/per visit) and a saliva sample for genetic testing ($5), totaling up to $185. Payment will be distributed via check or gift card at the end of all three visits.

### 2.4. Consent or assent

Once a referral is received, the MGH evaluation team will contact the student and their parent/guardian to seek written informed consent/assent prior to initiating any further study procedures. Once parent/legal guardian consent is obtained, eligible participants will provide assent following verbal and written explanation of the study, potential risks and voluntary nature of participation, right to withdraw, and details of data protection and confidentiality. Parental permission will not be required for students ages 18 and older.

### 2.5. Randomization

We will randomly allocate the timing of implementation of the iDECIDE program (vs. standard disciplinary response). Schools will be randomly phased into iDECIDE over the course of the trial (exposed phase; see Figure 1). When a school is randomized to iDECIDE, school staff will be trained as facilitators in the curriculum but will retain discretion as to which students should be enrolled in the program. To avoid risk of contamination bias within a school or district, school districts will serve as the unit of randomization with all schools within the same district randomized together. Randomization will occur by district, and stratified by (a) whether a district has any middle schools (yes/no) and (b) the number of total students in a district (low/high, using a median-split applied separately to the groups defined in (a) to ensure sufficient districts to distribute across waves). This stratification approach should help address differences in infraction rates for middle schools vs. high schools, and how smaller schools will, by necessity, have fewer potential participants. Cohorts 1 and 2 were randomized separately.

### 2.6. Blinding

Outcome assessors will be blinded to the school randomization phase. To avoid detection bias toward adjustments that favor statistical significance, initial implementation of analyses will also be “analyst-blind” (31). Only once data exclusion criteria have been finalized, models have been properly specified, and any other unforeseen circumstances have been addressed, will the unshuffled data be provided to the analyst for the final analysis implementation.

### 2.7. Interventions

#### 2.7.1. Discipline as usual

Standard disciplinary responses given by each partnering school will be used as the control during the unexposed phase of this trial. Standard responses may include detention, Saturday school, in-school suspension, out-of-school suspension, expulsion, citation, or any other punitive or non-punitive response given by schools per their existing policy at the point of a violation of school substance use policy. Students will report on the school response to the infraction during the baseline assessment.

#### 2.7.2. iDECIDE curriculum

Schools will be randomly phased into iDECIDE over the course of the trial (exposed phase; see Figure 1 and Section 2.5.).^1^ When a school is randomized to iDECIDE, school staff will be trained as facilitators in the curriculum but will retain discretion as to which students should be enrolled in the program.

iDECIDE promotes education and empowerment, instead of punishment, as an equitable response to adolescent substance use (1). Providing youth with science-based information and critical skills, iDECIDE challenges youth to make decisions that align with their core values, future goals, and support of their own personal wellbeing. While iDECIDE was designed as an ATP, it can be used outside of an infraction context as a targeted prevention for adolescents who are experimenting or are at risk for experimenting with substances. iDECIDE is intended to be slotted within tier 2 supports, or early indicated intervention, of school-based multi-tiered systems of support (32)—it is not a replacement for tier 1 universal prevention programming, nor is meant to be used in lieu of treatment or more intensive services when clinically indicated.

Curriculum development was guided by ongoing feedback from students and key school and community stakeholders. The iDECIDE curriculum is administered by a trained adult facilitator (see 2.7.2.1 below) in either a 1:1 or group setting. The curriculum consists of four core modules, each ~60–75-min in length. Module content covers teen brain development, neurobiology and addiction, industry tactics, risk and protective factors, drug effects, motives and triggers for use, healthy alternatives, mindfulness and meditation, core values, and goal setting (Table 1). The curriculum content is delivered through different modalities including educational videos, worksheets, handouts, group discussions, and on-your-own assignments. iDECIDE is drug agnostic, covering psychoeducational material relevant to alcohol, cannabis, nicotine, and other drugs. All information is hosted through an online learning management system. The curriculum, including all the supportive materail, is ADA-accessible and is available in English, Spanish, and Portuguese.

**Table 1:** Overview of the iDECIDE curriculum.

**Module**	**Name**	**Content**
1	Teen brain development, neurobiology and addiction, and industry tactics	Learn about neurobiology and addiction with a focus on teen brain development, industry tactics that target teens and other vulnerable populations, and their personal risk and protective factors for substance use and addiction.
2	Motives for use and specific drug effects	Learn about the neurobiology and effects of alcohol and other drugs on the brain and body. Assess substance use behavior and how it affects one's life.
3	Identifying triggers and healthy alternatives	Define and identify internal and external triggers to use alcohol and other drugs. Establish healthy alternatives to deal with urges to use substances by identifying realistic substance-free alternatives, developing effective communication strategies, and engage in mindfulness techniques.
4	Core values and setting goals	Identify core values, set short- and long-term goals that move toward overall wellness, and set actionable goal related to substance use that supports a healthier lifestyle and aligns with individual core values.

##### 2.7.2.1. Facilitator training

Individuals within each school will be identified to be trained as facilitators once a school has been randomized to transition from the unexposed to exposed phase of the study. Clinical training will not be required to serve as an iDECIDE facilitator. Facilitators will be required to attend a free, one-day, live training offered by MGH and MA DPH before gain access to any of the curriculum materials. During the training, facilitators will review the curriculum, gain access to the learning management system, and will cover other topics helpful in facilitating iDECIDE (e.g., motivational interviewing, managing unanticipated situations, and culturally responsive approaches). Facilitators will receive a copy of the facilitator manual and a participant workbook during their training. Each facilitator will be required to complete an annual fidelity check to maintain their certification as an iDECIDE facilitator. The fidelity check can be the option to re-attend a live training or have a designated iDECIDE team member observe and rate a live, recorded, or practice session of the curriculum.

### 2.8. Outcome measures

Assessments will occur at baseline (proximal to the point of the initial infraction), and ~45- and 90-days following baseline. Primary, secondary, and exploratory outcomes are described below. All measures, including descriptives and potential covariates, are listed in Table 2.

**Table 2:** Time and events.

**Assessments**	**Baseline (V1)**	**45-day follow-up (V2)**	**90-day follow-up (V3)**
**Primary outcomes**			
Knowledge of drug effects	X	X	X
Timeline follow-back	X	X^*^	X^*^
Urinalysis	X	X	X
**Secondary/exploratory outcomes**			
School climate measure	X	X	X
Infraction information survey	X	X	X
**Descriptions, effect modifiers, covariates**			
Demographics	X	-	-
Concomitant medications	X	X	X
Adverse events	-	X	X
MINI	X	-	-
C-SSRS	X	-	-
Participant psychiatric history	X	-	-
Family psychiatric history	X	-	-
UPPS-SP	-	-	-
AUDIT	X	-	-
CUDIT	X	-	-
FTND	X	-	-
ECDI	X	-	-
ASRI	X	-	-
MCQ delay discounting	X	-	-
Substance use history	X	-	-
Peer/partner substance use and tolerance of substance use	X	X	X
House rules about substance use	X	-	-

* means: a modified TLFB will be administered at V2 and V3 to gather substance use between visits.

#### 2.8.1. Primary outcome: knowledge of drug effects

The knowledge of drug effects will be operationalized as number of items correct on a custom-designed survey examining a student's understanding of the impact of alcohol, cannabis, nicotine, and other drugs on the brain and body, assessed at all three time points.

#### 2.8.2. Primary outcome: frequency of substance use

Substance use behavior will be assessed by how many days of the past 14 days a student spent using their preferred substance. Self-reported substance use will be assessed at all visits using the Timeline Followback (33, 34). A 90-day recall period will be queried at enrollment, and a modified Timeline Followback will be administered at subsequent visits to ascertain substance use in the period between visits.

Urine samples will be collected at all three visits to biochemically verify self-reported use. A 10-panel qualitative rapid dip drug test (Medimpex United Inc.) will be performed, qualitatively assessing for amphetamines (limit of quantitation [LOQ] = 1,000 ng/mL), cocaine (LOQ = 300 ng/mL), barbiturates (LOQ = 300 ng/mL), methamphetamines (LOQ = 1,000 ng/mL), benzodiazepines (LOQ 300 ng/mL), opiates (LOQ = 2,000 ng/mL), cannabinoids (THC) (LOQ 50 ng/mL), phencyclidine (LOQ = 25 ng/mL), oxycodone (LOQ = 100 ng/mL), and methadone (LOQ = 300 ng/mL). Quantitative urinary assays will also be completed at all visits (Dominion Diagnostics, North Kingstown, Rhode Island, USA). The quantitative assay includes creatinine-normalized 11-nor-9-carboxy-THC levels (CN–THCCOOH; LOQ: 5 ng/mg; upper limit of linearity: 500 ng/mg) using liquid chromatography–tandem mass spectrometry, and cotinine via enzyme immunoassay (EIA; LOQ: 500 ng/mL; upper limit of linearity: 2,000 ng/mL). Study staff will mail collection kits in advance of virtual visits to participants, and participants will provide the sample during the videoconferencing session. Qualitative results will be shown to the assessor during the visit, and kits will be shipped overnight for quantitative assays.

#### 2.8.3. Secondary outcome: feelings of school-based support

The secondary outcome will assess students' perception of quality of relationships with teachers/administrators, school connectedness, and social and emotional satisfaction at school. These school-based outcomes will be assessed with the emotional support form from the NIH toolbox emotion measures at all three time points (35, 36).

#### 2.8.4. Additional levels of data collection

Additional sources of data will be collected to explore how access to iDECIDE may impact changes in student- and school-level outcomes as well to define potential barriers to and supports of scalable implementation.

##### 2.8.4.1. Annual school-wide survey

In the fall semester of each academic year, schools will be asked to distribute a brief, de-identified survey during a school period, following an opt-out parental consent process. The purpose of the survey is to examine over time how implementation of iDECIDE impacts prevalence of substance use and perceptions of school as a supportive environment. Other domains queried in the survey include race and ethnicity, sexual identity and gender identity (37), perceived discrimination (38), and physical and emotional health (39–42).

##### 2.8.4.2. Pre-post curriculum survey

All students who participate in the iDECIDE curriculum will have the opportunity to complete a brief, anonymous survey immediately prior to the first curriculum session and immediately following the last curriculum session. Students will be provided a link to the pre- and post-survey by the trained iDECIDE facilitator. Domains queried include past seven days of substance use, perceived harm of substance use, knowledge of drug effects, and satisfaction with the curriculum.

##### 2.8.4.3. Key stakeholder interviews

At the point of randomization, schools will be given the opportunity to nominate three to five key school and community stakeholders. Nominated stakeholders will be (1) school faculty involved in responding to substance use related infractions and/or planning or delivery of social-emotional supports at participating school, or (2) community members (e.g., local coalition leaders, identified by the participating school to be trained in and deliver the iDECIDE intervention to school students). Stakeholders will complete a one-hour recorded semi-structured interview with a member of study staff ~two school months prior to being trained in iDECIDE and approximately six school months after implementation. Interviews will include prompts and questions to address beliefs, attitudes, and perceived effectiveness of substance use policies in their school, as well as structural factors that may contribute to transition to non-punitive responses to substance use in a school-based setting. A waiver of written consent was requested for this portion of the research protocol in which stakeholder participants will be provided a fact sheet detailing the interviews and verbal consent will be gathered at the beginning of each interview.

### 2.9. Confidentiality

Per standard school substance use policy, parents are notified by the school in the event of a substance use infraction. For this reason, parents will be aware of their student's substance use, and seeking consent to participate in the study will not inadvertently reveal student substance use habits to parents/guardians. Beyond basic eligibility criteria revealed in consent documents, no information will be shared with parents/guardians of participants under the age of 18 except when required by law (e.g., acute concern for safety of self or others; suspected child abuse). Confidentiality will be maintained by numerically coding all data and by keeping all data in password-protected, secure, HIPAA-compliant databases. All study staff will be trained in the protection of privacy of research participants and will have certification from the Collaborative Institutional Training Initiative. This study will maintain a Certificate of Confidentiality from the National Institutes of Health to protect against forced disclosure of identifiable, sensitive information collected as part of this clinical trial.

### 2.10. Data analysis

#### 2.10.1. Overarching statistical model

The two primary outcomes will be assessed at three time points: (1) immediately after a student is first caught for a substance use infraction (i.e., baseline, *time-of-infraction*); (2) 45 days after the date of the first infraction (i.e., *45 day-follow-up*); (3) 90 days after the date of the first infraction (i.e., *90 day-follow-up*). The proposed primary outcomes (items correct on a knowledge survey and number of days out of 14 spent using a preferred substance) are both bounded counts variables. We therefore will assume (a) whether an item is correct on the knowledge survey follows a Bernoulli distribution and the number of days out of 14 spent using a substance follows a binomial distribution, and (b) these distributions (both the binomial and Bernoulli) are governed by a latent probability parameter. This latent probability describes either the likelihood of getting an item correct on the knowledge survey or the average number of days spent using a preferred substance out of 14, and the log-odds for a student's latent probability at the three assessment time points is described following a trivariate normal distribution. This statistical model will allow us to examine changes in substance use and knowledge about substances while controlling for (1) measurement error, and (2) correlations between repeated measures collected over the three time points. The means for the log-odds of the latent probabilities can be further decomposed into a summation of population-level, school-level, and student-level effects. The specific decomposition of the means for the log-odds will depend on the outcome measure.

The complexity of the statistical models (e.g., a multivariate latent measure model with both fixed and random effects and with observed data distributed as counts) is most easily handled within a Bayesian framework. However, prior distributions will need to be defined for model parameters. A conservative approach will be taken using only weakly informative priors with a diffuse range (for example, standard deviations for log-odds will be set to 2.5, estimates for fixed effects will be centered at 0, and we will use empirical Bayes priors for intercepts).

#### 2.10.2. Model specifics—knowledge of drug effects

The average log-odds for probability of obtaining an item correct at each time point will be further decomposed by including, at a minimum: (1) a student-varying intercept to control for individual differences in substance use, (2) an item-varying intercept controlling for differences in difficulty between questions, (3) a school-varying intercept controlling for differences in policy in disciplinary actions and substance use culture across schools, and (4) fixed effects implementing a quadratic time trend over the number of months since the start of the study. If necessary, additional terms (i.e., an interaction between time point and intervention or covariates) can be incorporated.

#### 2.10.3. Model specifics—frequency of substance use

The average log-odds for probability of use at each time point will be further decomposed by including, at a minimum: (1) a student-varying intercept to control for individual differences in substance use, (2) a school-varying intercept controlling for differences in policy in disciplinary actions and substance use culture across schools, (3) fixed effects implementing a quadratic time trend over the number of months since the start of the study, and (4) fixed effects describing differences in days spent using over the three types of substances (alcohol, cannabis, and nicotine). Additional terms (i.e., an interaction between time point or substance type and intervention or covariates) can be incorporated as necessary.

#### 2.10.4. Effect of iDECIDE

The primary effects of interest are whether, for students assigned to the iDECIDE program after their first substance use infraction vs. students who receive standard disciplinary actions, there is (a) an increase in knowledge about substances and their impact and (b) a reduction in days spent using. Effects will be examined using a fixed effect coded as 0 at the *time-of-infraction* and for follow-up time points pre-intervention and coded as 1 at the *45-day-follow-up* and *90-day-follow-up* time points post-intervention. The primary analysis will be the interaction of this variable with a variable indicating whether the school district is in the exposed phase of the study.

We will use an intent-to-treat design. We will examine any student with a substance use infraction in the exposed phase irrespective of actual attendance in the program. Data for primary analyses will only be considered from the first infraction on record.

The study will be conducted as (a) a superiority study on the knowledge endpoint, and (b) a non-inferiority trial on the substance use endpoint. Statistical significance will be defined as a two-sided posterior *p* < 0.025. The non-inferiority margin for substance usage was determined via the fixed margin method (43). Evan-Whipp et al. (44) report results from the International Youth Development Study on what school discipline policies predict reductions in cannabis use. Two discipline approaches significantly predicted a reduction in cannabis use: lecture from teacher (OR = 0.61, 95% CI = 0.45 to 0.83) and police involvement (OR = 0.74, 95% CI = 0.55 to 1.00). The pooled results (OR = 0.68, 95% CI = 0.50 to 0.92) suggest a lower margin of 1.09. Corresponding analyses for alcohol (45) and cigarette use (46) found no significant discipline approaches that predicted reduced use after adjusting for confounders. Using a preserved effect of 50% per FDA guidelines, the minimum non-inferiority threshold based on previous literature is an odds ratio of 1.045.

#### 2.10.5. Clinical significance

The direction and statistical significance for the difference in outcomes prior vs. after implementation of the iDECIDE program yield the following possible results for each outcome: (1) The iDECIDE program results in changes in the knowledge survey that are either (a) superior, (b) inferior, or (c) non-significant compared to standard disciplinary approaches, (2) the iDECIDE program results in reduction in substance use that is either (a) superior, (b) equivalent, (c) non-inferior, or (d) inferior compared to standard disciplinary approaches. The resulting 12 combinations of the results for the knowledge survey and substance use can be partitioned into 3 decisions regarding the clinical significance of the iDECIDE program compared to standard disciplinary approaches: (1) as or better performance, (2) worse performance, or (3) complex results (e.g., improvement in one outcome combined with worse performance on the other). Table 3 summarizes the 12 possible combinations of results and the corresponding decision about the effectiveness of the iDECIDE curriculum.

**Table 3:** Pattern of significant finding and associated conclusion regarding relative effectiveness of iDECIDE vs. standard disciplinary action.

Significant change relative to standard disciplinary actions^*^	**Conclusion**
Substance use ▾ and knowledge score ▴	iDECIDE program is as or more effective than standard disciplinary actions.
Substance use ■ and knowledge score ▴	
Substance use ▾ and knowledge score ▴	
Substance use ▾ and knowledge score ~	
Substance use ■ and knowledge score ~	
Substance use ▾ and knowledge score ~	
Substance use ▴ and knowledge score ▾	iDECIDE program is less effective than standard disciplinary actions.
Substance use ▴ and knowledge score ~	
Substance use ▴ and knowledge score ▴	Pattern of results is complex and difficult to interpret, requiring further study.
Substance use ▾ and knowledge score ▾	
Substance use ■ and knowledge score ▾	
Substance use ▾ and knowledge score ▾	

* Statistical significance defined as p < 0.025 for a two-tailed test; The ▾ and ▴ symbols indicate a significant improvement in knowledge/drug use relative to standard disciplinary action (Superiority), the ▾ and ▴ symbols indicate a significant decrement relative to standard disciplinary action (Inferiority), and the ■ and ▾ symbols indicate equivalence and non-inferiority, respectively, relative to standard disciplinary action (specific to substance use), and the ~ symbol indicates no statistically significant difference (p > 0.025) between the iDECIDE program and standard disciplinary action (specific to the knowledge survey).

#### 2.10.6. Covariates

The covariates we will consider, at a minimum, are: (a) two terms implementing a quadratic trend for number of months since the start of the program, (b) a student's preferred substance (i.e., alcohol, cannabis, or nicotine), (c) a student's baseline level of dependence for his or her primary substance (based on appropriate self-report questionnaires), (d) number of repeat substance use infractions that a student has, (e) number of peers who use drugs, (f) a student's age at baseline, (g) a student's gender (or if cell counts are too low, biological sex), and (h) whether school is urban, rural, or suburban. These covariates will be used as part of missing data imputation, and if necessary, may be included in the primary analysis (to be determined during the analyst-blind period). If any additional covariates will be deemed necessary for inclusion, this will be done before the blind is broken for the analyst.

#### 2.10.7. Missing data

Analysis of the primary outcome will require students to provide a pair of observations and the number of days spent using a substance at both time-of-infraction and post-discipline time points. If there is loss to follow-up, there then is the risk that a pairwise deletion of data points will (a) lead to a substantial reduction in sample size, and (b) risk bias in estimates if loss to follow-up is systematically different between those undergoing standard disciplinary actions vs. those enrolled in the iDECIDE program. Missing data will therefore be handled via multivariate imputation by chained equations (47). Values for missing observations will be imputed 40 times using baseline sample characteristics: (a) a student's preferred substance (i.e., alcohol, cannabis, or nicotine), (b) a student's baseline level of dependence for his or her primary substance (based on appropriate self-report questionnaires), (c) number of repeat substance use infractions that a student has, (d) number of peers who use drugs, (e) student's age at baseline, (f) a student's gender (or if cell counts are too low, biological sex), and (g) whether school is urban, rural, or suburban. The analyses will be rerun for each set of imputations and results will be pooled by combining the posterior draws across imputations to obtain appropriate estimates and measures of uncertainty.

#### 2.10.8. Inverse probability weights

The longitudinal aspect of the study design, combined with an inherent possibility of treatment contamination, results in a risk that sample characteristics may differ between the start of the study and the end of the study. For example, in the initial months of the program, the sample will consist of a mix of students more and less responsive to the intervention. However, in later months, students who would be more responsive to the intervention but have not yet committed an infraction could be influenced by students who already received the intervention, and thereby become less likely to commit infractions in the first place. In this scenario, sample characteristics will thereby shift in later months to consist more of less responsive students, resulting in biased estimates that do not properly reflect the efficacy of the study.

We can correct for potential bias due to differing sample characteristics via inverse probability weights (48). Note, however, that standard methods for inverse probability weights (IPW) do not translate easily to Bayesian settings. Therefore, we will either fully implement the two-step method proposed by Liao and Zigler (49), or if computational challenges make this infeasible, implement a pseudo-Bayesian approach. The first step will be to compute the IPW by re-administering a custom school-wide survey (used as part of screening) to students who are enrolled in the study. We will fit a logistic regression predicting referral status (pre- vs. post-intervention) for each student using the following predictors from the survey: (1) whether a student is an athlete (yes or no), (2) a student's grade-point average, (3) a student's post-graduation plans (four-year college, two-year college, trade school, military, getting a job, unknown, and an “other” category), (4) prior suspensions (none, drug-related, and other), (5) race, and (6) ethnicity.

We will then compute stabilized weights per subject (i.e., the unconditional probability of referral divided by the probability of referral conditioned on the predictors from the school-wide survey) per each posterior draw. The second step will be to use the stabilized weights to adjust the likelihood for the statistical model used with the primary analysis. This can either be done (1) in a fully Bayesian fashion, using the full set of stabilized weights computed per each posterior draw from step one, or, if this proves computationally infeasible, (2) in a pseudo-Bayesian fashion, using the weight per subject averaged over posterior draws (i.e., maximum a posteriori estimates for weights). The likelihood used for sampling from the posterior will be re-weighted by the subject-level weights to adjust for potential confounding due to differing sample composition.

#### 2.10.9. Model specifics for secondary outcomes

Change in quality of relationships with teachers/administrators, school connectedness, social and emotional satisfaction at school (36) will be evaluated as secondary outcomes. Summed scores from the emotional support form will be assumed to follow a binomial distribution, analyzed with an equivalent statistical model, and used for the number of days spent using a preferred substance.

#### 2.10.10. Sensitivity analyses

For all outcomes, we will conduct at least five sensitivity analyses. We will examine how robust our conclusions are to implementation of the intent-to-treat approach and also conduct an as-treated analysis, restricting comparisons for the key effect to students confirmed to have received standard disciplinary action pre-intervention vs. students confirmed to have attended iDECIDE sessions post-intervention. We will examine how robust our conclusions are to the assumption of additivity with the quadratic time trend and will fit an interaction model to examine (a) the statistical significance of the interaction terms between the key comparison and the quadratic time trend, and (b) the difference in performance on the leave-one-out cross-validation (LOO-CV) approximation between the additive and interaction models. We will examine how robust our conclusions are to the assumption of additivity with the three types of substances (alcohol, nicotine, and cannabis) and will again fit an interaction model and examine (a) the statistical significance of the interaction terms between the key comparison and the categorical predictors for substance type, and (b) the difference in performance on the LOO-CV approximation between the additive and interaction models. We will examine how robust our conclusions are to our missing data approach, rerunning the primary analysis using only observed data. Finally, we will examine how robust our conclusions are to implementation of the IPW approach, rerunning the primary analysis without inclusion of the stabilized weights.

#### 2.10.11. Enrollment of additional schools

It is possible that additional schools will enroll in the iDECIDE program post-randomization (for example, a school may choose to enroll in the second year of the study). Schools that enroll after the initial randomization will be excluded from primary analyses, as randomization for schools that enroll later will result in especially skewed distributions across waves (there will be fewer waves for schools that enroll later to be randomized across). However, assuming sufficient schools enroll post-randomization, we will conduct a secondary analysis to examine whether conclusions change when including schools that enroll late in the program.

#### 2.10.12. Power

We determined power using the R package “swCRTdesign” (50) which was explicitly designed to estimate power for stepped wedge designs. We were able to power the study using student enrollment data and publicly available data on 2018–2019 drug infraction rates (51) for the 67 schools who agreed to participate as part of Cohort 1. There were 16 schools without infraction rate data. For simplicity, this missing data was imputed by examining 522 schools with complete data, identifying schools that matched in type (e.g., middle school, high school, etc.) and had the closest number of students. Infraction rates from this subset of schools of the same type and equivalent student size were then substituted for the missing values.

The number of participants per cluster will not depend on the schools assigned to the cluster, but rather the number of students in the schools who will have a substance use infraction. We cannot know the exact number in advance, but we can simulate the predicted number of participants via a binomial distribution using student enrollment for the upcoming 2 years and the infraction percentages from 2018 to 2019. Following the proposed stratified randomization scheme we will use in the actual study, schools were assigned to each cluster at the district-level, stratified by whether a district had any middle schools (yes/no) and school size (using a median split of 1,460 for districts with middle schools and 829 for those without). This approach for generating sample sizes was repeated 100 times to integrate over uncertainty and the final estimate of power was obtained by averaging over the repetitions. As noted earlier, to determine the number of waves to use, we examined power assuming four waves, seven waves, and 10 waves.

Additional parameters required for power estimation were the means in the unexposed and exposed study phases and the pooled standard deviation. For simplicity, means were based on the ratio of the proportion of days spent using a drug at the follow-up time point divided by the proportion of days spent using a drug at the baseline visit. This format allowed easy specification of a clinically meaningful effect, comparing an unexposed ratio of 1 (no change) to an exposed ratio of 0.8 (a 20% reduction in use). To estimate the pooled standard deviation for the ratio, we used data collected in a previous study (NCT: 03276221) from 63 high school students, examining (1) the number of days spent using cannabis over a 30-day interval before a baseline visit, and (2) the number of days spent using cannabis before a 7-week visit over an average monitoring period of 22 days (SD of 10 days). The standard deviation for the ratio of the proportion of days spent before the 7-week visit over the proportion of days spent before the baseline visit was 0.79. Finally, values for the intra-class correlation (ICC) and the cluster auto-correlation (CAC) were required. Based on a large meta-analysis (52), we fixed the ICC and CAC to 0.02 and 0.22, respectively. Power was estimated as 0.75 (SD = 0.03) for 4 waves, 0.82 for 7 waves (SD = 0.03), and 0.84 for 10 waves (SD = 0.03).

#### 2.10.13. Software

All analyses will be conducted using the statistical software R (version 4.1.1) (53) and integrated development environment RStudio (54). Data will be prepared using the R packages “dplyr” (55) and “tidyr” (56). Bayesian analyses will be done using the R packages “rstan” (57) and “brms” (58) Missing data will be imputed using the R package “mice” (47) Reproducible code and de-identified data will be organized using the R package “targets” (59).

## 3. Trial status

This study is ongoing and currently recruiting participants from partnering schools. At the time of submission of this manuscript, 122 participants have been enrolled. The first student enrollment occurred in February 2022.

## 4. Discussion

While punishment for school-based substance use infractions are now recommended only as a last resort by the Federal Department of Education, there are no existing rigorous trials that establish an evidence base of curricula to be delivered as ATP. It is imperative to develop and evaluate the effectiveness of diversion programs and other novel school-based interventions to better identify and implement programming specific to the needs of students across a variety of school and district settings. We will conduct a pragmatic clinical effectiveness trial to evaluate the iDECIDE curriculum, utilizing a stratified randomization scheme and implementing the program approximately every two to three school months over approximately a 36-month period.

This will be the first study of its kind to evaluate a promising diversion program, used as an alternative to punishment for school-based substance-use infractions. We hypothesize that student-level outcomes will improve when schools transition from a standard disciplinary response to having access to a more educational and therapeutic alternative.

## Ethics statement

The studies involving humans were approved by Mass General Brigham (MGB) Institutional Review Board (IRB). The studies were conducted in accordance with the local legislation and institutional requirements. Written informed consent for participation in this study was provided by the participants' legal guardians/next of kin. No potentially identifiable images or data are presented in this study.

## Author contributions

This study was conceived and designed by RS, with support for implementation design by RB, GP, KP, CG, VI, BD, MP, AB, DM, and JW, who made relevant contributions to the study design and procedure. AT, SL, and JW were instrumental in the development and review of the diversion program and have continued to provide feedback on all implementation and study related procedures. All authors read the manuscript, made significant contributions, and approved the final version.

## Footnotes

^1^http://www.iDECIDEmyfuture.org; https://www.youtube.com/watch?v=vwSLBmX32Mg

## References

[1] Miech RA Johnston LD Patrick ME O'Malley PM Bachman JG Schulenberg JE. Monitoring the Future National Survey Results on Drug Use, 1975-2022: Secondary School Students. Ann Arbor, MI: Institute for Social Research, The University of Michigan. (2023).

[2] Student, Discipline Statewide Report. 2021-22 Student Discipline Statewide Report–All Offenses–All Students. Available online at: https://profiles.doe.mass.edu/statereport/ssdr.aspx (accessed, 2022).

[3] Tavakolian HR Howell N. Dropout dilemma and interventions. Global Educ J. (2012) 1:77–81.

[4] Dudovitz RN McCoy K Chung PJ. At-school substance use as a marker for serious health risks. Acad Pediatr. (2015) 15:41–6. 10.1016/j.acap.2014.06.022 25528124 PMC4273105

[5] Hemphill SA Heerde JA Herrenkohl TI Toumbourou JW Catalano RF. The impact of school suspension on student tobacco use: a longitudinal study in Victoria, Australia, and Washington State, United States. Health Educ Behav. (2012) 39:45–56. 10.1177/1090198111406724 21586667 PMC3158957

[6] Hemphill SA Herrenkohl TI Plenty SM Toumbourou JW Catalano RF McMorris BJ. Pathways from school suspension to adolescent nonviolent antisocial behavior in students in Victoria, Australia and Washington State, United States: pathways from school suspension to antisocial behavior. J Community Psychol. (2012) 40:301–18. 10.1002/jcop.20512 24049218 PMC3774047

[7] Truth Initiative. Discipline Is Not the Answer: Better Approaches to On-campus Student Tobacco Use. Available online at: https://truthinitiative.org/research-resources/emerging-tobacco-products/discipline-not-answer

[8] Kelly AB Evan-Whipps T Smith R Chan G Toumbourou JW Patton GC . A longitudinal study of the association of adolescent polydrug use, alcohol use and high school non-completion. Addiction. (2015) 110:627–35. 10.1111/add.12829 25510264 PMC4361375

[9] Arcia E. Achievement and enrollment status of suspended students: outcomes in a large, multicultural school district. Educ Urban Soc. (2006) 38:359–69. 10.1177/0013124506286947

[10] Noltemeyer AL Ward RM Mcloughlin C. Relationship between school suspension and student outcomes: a meta-analysis. School Psych Rev. (2015) 44:224–40. 10.17105/spr-14-0008.134117607

[11] Skiba RJ Rausch MK. Zero tolerance, suspension, and expulsion: Questions of equity and effectiveness. In: Handbook of classroom management. London: Routledge (2013) p. 1073–1100.

[12] Jones EP Margolius M Rollock M Yan CT Cole ML Zaff JF. Disciplined and disconnected: how students experience exclusionary discipline in minnesota and the promise of non-exclusionary alternatives. In: America's Promise Alliance. Washington, DC (2018).

[13] Mendez LMR Knoff HM. Who gets suspended from school and why: a demographic analysis of schools and disciplinary infractions in a large school district. Educ Treat Child. (2003) 26:30–51. Available online at: https://www.jstor.org/stable/42900535

[14] Finn KV Willert HJ. Alcohol and drugs in schools: teachers' reactions to the problem. Phi Delta Kappan. (2006) 88:37–40. 10.1177/003172170608800108

[15] Balfanz R Fox J. Sent home and put off-track: the antecedents, disproportionalities, and consequences of being suspended in the ninth grade. J Appl Res Child. (2014) 5:13. 10.58464/2155-5834.1217

[16] Hemphill SA Plenty SM Herrenkohl TI Toumbourou JW Catalano RF. Student and school factors associated with school suspension: a multilevel analysis of students in Victoria, Australia and Washington State, United States. Child Youth Serv Rev. (2014) 36:187–94. 10.1016/j.childyouth.2013.11.022 24860205 PMC4028069

[17] Okonofua JA Walton GM Eberhardt JL A. vicious cycle: a social–psychological account of extreme racial disparities in school discipline. Perspect Psychol Sci. (2016) 11:381–98. 10.1177/1745691616635592 27217251

[18] Blum R. School Connectedness: Improving the Lives of Students. Baltimore, MD (2005).

[19] National Association of School Psychologists. Zero tolerance and Alternative Strategies: A Fact Sheet for Educators and Policymakers. (2008). Available online at: http://www.nasponline.org/resources/factsheets/zt_fs.aspx (accessed, 2008).

[20] Benson PL Scales PC Hamilton SF Sesma A. Positive youth development: theory, research, and applications. In: Handbook of Child Psychology. Baltimore, MD (2006).

[21] Liu J Butler R Turncliff A Gray C Lynch S Whittaker J . (in press). An urgent need for school-based diversion programs for adolescent substance use: a statewide survey of school personnel. J Adolesc Health. (2023). 10.1016/j.jadohealth.2023.04.006 37318411 PMC10524742

[22] US Department of Education. 2015–2016 Civil Rights Data Collection: School Climate and Safety. Washington, DC (2018).

[23] Curran GM Bauer M Mittman B Pyne JM Stetler C. Effectiveness-implementation hybrid designs: combining elements of clinical effectiveness and implementation research to enhance public health impact. Med Care. (2012) 50:217–26. 10.1097/MLR.0b013e3182408812 22310560 PMC3731143

[24] Proctor E Silmere H Raghavan R Hovmand P Aarons G Bunger A . Outcomes for implementation research: conceptual distinctions, measurement challenges, and research agenda. Adm Policy Ment Health. (2011) 38:65–76. 10.1007/s10488-010-0319-7 20957426 PMC3068522

[25] Moher D. The CONSORT statement: revised recommendations for improving the quality of reports of parallel-group randomized trials. JAMA. (2001) 285:1987. 10.1001/jama.285.15.1987 11308435

[26] Moher D Schulz KF Altman DG. The CONSORT statement: revised recommendations for improving the quality of reports of parallel-group randomised trials. Lancet. (2001) 357:1191–4. 10.1016/S0140-6736(00)04337-3 11323066

[27] Moher D Schulz KF Altman DG and for the CONSORT Group*. The CONSORT statement: revised recommendations for improving the quality of reports of parallel-group randomized trials. Ann Intern Med. (2001) 134:657. 10.7326/0003-4819-134-8-200104170-00011 11304106

[28] Moher D Schulz KF Altman DG. The CONSORT statement: revised recommendations for improving the quality of reports of parallel group randomized trials. BMC Med Res Methodol. (2001) 1:2. 10.1186/1471-2288-1-2 11336663 PMC32201

[29] Chan A-W Tezlaff JM Altman DG Laupacis A Gøtzsche PC Krleža-Jerić K . SPIRIT 2013 statement: defining standard protocol items for clinical trials. Ann Intern Med. (2013) 158:200. 10.7326/0003-4819-158-3-201302050-00583 23295957 PMC5114123

[30] Chan A-W Tezlaff J Gøtzsche PC Altman D Mann H Berlin J . SPIRIT 2013 explanation and elaboration: guidance for protocols of clinical trials. BMJ. (2013) 346:e7586–e7586. 10.1136/bmj.e7586 23303884 PMC3541470

[31] Dutilh G Sarafoglou A Wagenmakers E-J. Flexible yet fair: blinding analyses in experimental psychology. Synthese. (2021) 198:5745–72. 10.1007/s11229-019-02456-7

[32] DESE. Multi-Tiered System of Support (MTSS)–Systems for Student Success Office. Available online at: https://www.doe.mass.edu/sfss/mtss/ (accessed, 2020).

[33] Sobell L Sobell M Buchan G. Timeline Followback Method (Drugs, Cigarettes, and Marijuana). Lauderdale, FL (1996).

[34] Robinson SM Sobell LC Sobell MB Leo GI. Reliability of the Timeline Followback for cocaine, cannabis, and cigarette use. Psychol Addict Behav. (2014) 28:154–62. 10.1037/a0030992 23276315

[35] Paolillo EW . NIH Toolbox^®^ emotion batteries for children: factor-based composites and norms. Assessment. (2020) 27:607–20. 10.1177/1073191118766396 29618218 PMC6205918

[36] Babakhanyan I McKenna BS Casaletto KB Nowinski CJ Heaton RK. National institutes of health toolbox emotion battery for english- and spanish-speaking adults: normative data and factor-based summary scores. Patient Relat Outcome Meas. (2018) 9:115–27. 10.2147/PROM.S151658 29588623 PMC5859895

[37] Srinivasan S Goldhammer H Crall C Kitts R Keuroghlian AS. A novel medical student elective course in lesbian, gay, bisexual, transgender, queer, intersex, asexual, and sexually and gender diverse health: training tomorrow's physician-leaders. LGBT Health. (2022) 10:252–7. 10.1089/lgbt.2022.0161 36350692

[38] Fisher CB Wallace SA Fenton RE. Discrimination distress during adolescence. J Youth Adolesc. (2000) 29:679–95. 10.1023/A:1026455906512

[39] Kelleher C. Minority stress and health: Implications for lesbian, gay, bisexual, transgender, and questioning (LGBTQ) young people. Couns Psychol Q. (2009) 22:373–9. 10.1080/09515070903334995

[40] Kelleher I Harley M Murtagh A Cannon M. Are screening instruments valid for psychotic-like experiences? A validation study of screening questions for psychotic-like experiences using in-depth clinical interview. Schizophr Bull. (2011) 37:362–9. 10.1093/schbul/sbp057 19542527 PMC3044617

[41] Nock MK Holmberg EB Photos VI Michel BD. Self-injurious thoughts and behaviors interview: development, reliability, and validity in an adolescent sample. Psychol Assess. (2007) 19:309–17. 10.1037/1040-3590.19.3.309 17845122

[42] Nock MK Wedig MM Holmberg EB Hooley JM. The emotion reactivity scale: development, evaluation, and relation to self-injurious thoughts and behaviors. Behav Ther. (2008) 39:107–16. 10.1016/j.beth.2007.05.005 18502244

[43] Research C. for D. E. and. Non-Inferiority Clinical Trials. US Food and Drug Administration. Available online at: https://wwwfdagov/regulatory-information/search-fda-guidance-documents/non-inferiority-clinical-trials (accessed, 2020).

[44] Evans-Whipp TJ Plenty SM Catalano RF Herrenkohl TI Toumbourou JW. Longitudinal effects of school drug policies on student marijuana use in Washington State and Victoria, Australia. Am J Public Health. (2015) 105:994–1000. 10.2105/AJPH.2014.302421 25790384 PMC4386529

[45] Evans-Whipp TJ Plenty SM Catalano RF Herrenkohl TI Toumbourou JW. The impact of school alcohol policy on student drinking. Health Educ Res. (2013) 28:651–62. 10.1093/her/cyt068 23766454 PMC3708139

[46] Evans-Whipp TJ Bond L Ukoumunne OC Toumbourou JW Catalano RF. The impact of school tobacco policies on student smoking in Washington State, United States and Victoria, Australia. Int J Environ Res Public Health. (2010) 7:698–710. 10.3390/ijerph7030698 20616998 PMC2872326

[47] Buuren S. van and Groothuis-Oudshoorn K. mice: multivariate imputation by chained equations in R. J Stat Soft. (2011) 45:i03. 10.18637/jss.v045.i03

[48] Robins JM Hernan MA Brumback B. Marginal structural models and causal inference in epidemiology. Epidemiology. (2000) 11:550–560. 10.1097/00001648-200009000-00011 10955408

[49] Liao SX Zigler CM. Uncertainty in the design stage of two-stage Bayesian propensity score analysis. Stat Med. (2020) 39:2265–90. 10.1002/sim.8486 32449222 PMC9170228

[50] Hughes J Hakhu N Voldal E. Stepped Wedge Cluster Randomized Trial (SW CRT) Design. (2019).

[51] Massachusetts Department of Elementary and Secondary Education. Student Discipline Data Report–Illegal Substances. (2012).

[52] Korevaar E Kazsa J Taljaard M Hemming K Haines T Turner EL . Intra-cluster correlations from the CLustered OUtcome Dataset bank to inform the design of longitudinal cluster trials. Clin Trials. (2021) 18:529–40. 10.1177/17407745211020852 34088230

[53] R Core Team. R: A Language and Environment for Statistical Computing (version 4.1.1). Vienna: R Foundation for Statistical Computing. (2018).

[54] RStudio Team. RStudio: Integrated Development Environment for R. Boston, MA (2021).

[55] Wickham H Francois H Muller K. dplyr: A Grammar of Data Manipulation (2023).34028547

[56] Wickham H Vaughan D Girlich M. tidyr: Tidy Messy Data. Boston, MA (2023).

[57] Stan Development Team. RStan: The R Interface to Stan. Dortman (2020).

[58] Burkner P. brms: Bayesian Regression Models using ‘Stan'. Indianapolis, IN (2017).

[59] Landau W. The targets R package: a dynamic Make-like function-oriented pipeline toolkit for reproducibility and high-performance computing. JOSS. (2021) 6:2959. 10.21105/joss.02959

